# Supplementary material for: Identification of photoperiod-induced specific miRNAs in the adrenal glands of Sunite sheep (Ovis aries)
Source: Front Vet Sci. 2022 Jul 22;9:888207. doi: 10.3389/fvets.2022.888207 (PMC9354845; doi:10.3389/fvets.2022.888207)
Supplement: Supplementary file 1 [file Data_Sheet_1.ZIP › Supplementary file/miRDeep2 file.zip!/miRDeep2 file/Novel_Structure/SPLP42A2/Novel_684.pdf]

Provisional ID	:	NC_019477.2_35683
Score total	:	1.6
Score for star read(s)	:	-1.3
Score for read counts	:	-0.8
Score for mfe	:	2.2
Score for randfold	:	1.6
Score for cons. seed	:	
Total read count	:	10
Mature read count	:	9
Loop read count	:	0
Star read count	:	1

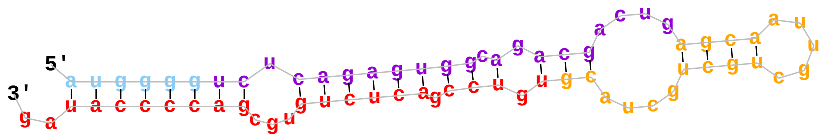

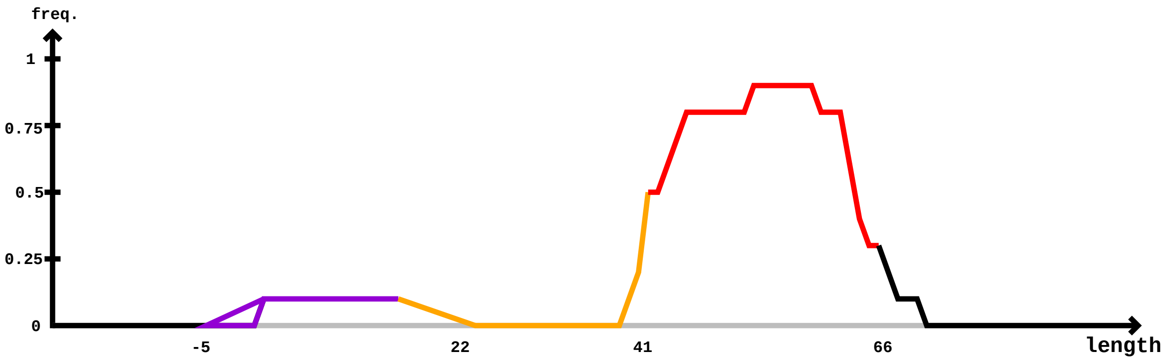

Star **Mature**

```
5'- gcggguauaguccauggggucucagaguggcagacgacugagcaauugcugcugcuacguguccgacucugugcgaccccauagacggcagcccgccaggcucccccguc   -3'    obs
    gcggguauaguccauggggucucagaguggcagacgacugagcaauugcugcugcuacguguccgacucugugcgaccccauagacggcagcccgccaggcucccccguc          exp
    (((((.....(((.((((((((.((((((((.(.(((....((((.....))))....))).))).))))))...)))))))).)))((.((((.....))))))))))).   reads      mm        sample
    ...................ucucagaguggcagacgacug......................................................................   1          0         seq
    .........................................................cguguccgacucugugcgac.................................   1          0         seq
    ..........................................................guguUcgacucugugcgacccc..............................   1          1         seq
    ...........................................................uguccgacucugugcgacccc..............................   1          0         seq
    ...........................................................uguccgacucugugcgacccca.............................   1          0         seq
    ...........................................................uguccgacucugugcgaccccauaA..........................   1          1         seq
    .............................................................uccgacucugugcgaccccCu............................   1          1         seq
    ..............................................................ccgacucugugcgacccca.............................   1          0         seq
    ...............................................................cgacucugugcgaccAcauag..........................   1          1         seq
    ......................................................................gugcgaccccauagacgA......................   1          1         seq
```
